# Tracking Adaptive Pathways of Invasive Insects: Novel Insight from Genomics

**DOI:** 10.3390/ijms24098004

**Published:** 2023-04-28

**Authors:** Zhongxiang Sun, Yao Chen, Yaping Chen, Zhihui Lu, Furong Gui

**Affiliations:** State Key Laboratory of Conservation and Utilization of Biological Resources of Yunnan, College of Plant Protection, Yunnan Agricultural University, Kunming 650201, China

**Keywords:** whole-genome sequencing, invasive insects, invasion mechanism, prevention and control technology

## Abstract

Despite the huge human and economic costs of invasive insects, which are the main group of invasive species, their environmental impacts through various mechanisms remain inadequately explained in databases and much of the invasion biology literature. High-throughput sequencing technology, especially whole-genome sequencing, has been used as a powerful method to study the mechanisms through which insects achieve invasion. In this study, we reviewed whole-genome sequencing-based advances in revealing several important invasion mechanisms of invasive insects, including (1) the rapid genetic variation and evolution of invasive populations, (2) invasion history and dispersal paths, (3) rapid adaptation to different host plant ranges, (4) strong environmental adaptation, (5) the development of insecticide resistance, and (6) the synergistic damage caused by invasive insects and endosymbiotic bacteria. We also discussed prevention and control technologies based on whole-genome sequencing and their prospects.

## 1. Introduction

Invasive alien species (IASs), which are species introduced from an exotic origin, cause harm to the invaded ecosystems; they have a variety of superior invasive properties that enable them to outperform native species [1]. IASs are a primary threat to global biodiversity, economies, agriculture, and human health [2,3]. Invasions involving insects, which are the main group of invasive species (accounting for approximately 35%), are one of the major challenges facing the stability and development of the global ecosystem [4,5]. The damage caused by invasive insects costs at least USD 70 billion per year globally, and the associated annual health costs exceed USD 6.9 billion [6]. As the most notable invasive insect in recent times, the fall armyworm (FAW), *Spodoptera frugiperda*, which originated from tropical and subtropical areas of America more than 100 years ago, has caused devastating damage to corn and other crops in Africa and China since its first appearance in early 2016 and 2019, respectively [7,8,9]. The invasive and destructive South American tomato pinworm, *Tuta absoluta* (Meyrick), is on an invasion journey eastward, successfully invading most Asian countries since its initial detection in Turkey in 2009, leading to extensive damage to tomato production [10]. In addition to the direct damage to agriculture, invasive insects further cause additional and unpredictable harm to human health and sustainable economies. For instance, the brown marmorated stink bug (*Halyomorpha halys*) indirectly impacts the fumonisin contamination of corn fields in the USA [11], and the Asian tiger mosquito (*Aedes albopictus*), the vector of dengue virus, has spread throughout South America [12].

As the most pervasive invasive species, alien invasive insects have the propensity to evolve more rapidly than non-invasive species, and this makes them better able to colonize novel habitats and even occupy novel niches [13,14]. Invasive insects harm the environment through various mechanisms, including their superior migratory and spreading abilities [15], rapid adaptation to host plants [16], and the development of better detoxification and adaptation mechanisms to xenobiotics such as insecticides [17]. Although the many problems caused by invasive insects are due to a variety of reasons (including international trade, plant quarantine failures, public ignorance, and so on) [4], systematic and in-depth studies of invasive biology and the invasion mechanisms of invasive insects are crucial to the development of management strategies for further monitoring and control. However, despite their series of ecological impacts, relatively few studies have been conducted on invasive insect species [18].

To fully understand the molecular evolution, gene composition, and expression regulation of invasive insects, high-throughput sequencing of the whole genome of invasive species is required. High-throughput sequencing technology allows hundreds of thousands or even millions of DNA molecules to be simultaneously sequenced. The introduction of this technology has enabled the mining of genetic polymorphisms and gene functions at the genome level. With its development, the cost of high-throughput sequencing has gradually decreased, and it has been used for different invasive species to analyze and understand the biology of invasion. Here, we review the recent advances in revealing the invasion mechanisms of invasive insects by using whole-genome sequencing.

## 2. Whole-Genome Sequencing of Invasive Insects

**Figure 1:**
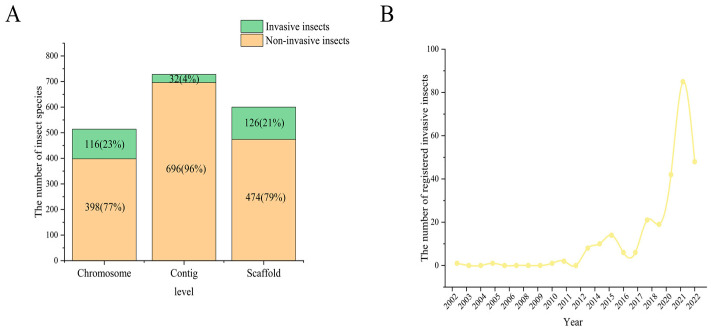
Statistics of registered insect genomes at NCBI. (**A**) Number of genome assemblies of insects and invasive insects registered with the National Center for Biotechnology Information. (**B**) Number of invasive insect genomes at the time of release.

As of March 2023, 1842 genome assemblies of insects have been registered with the National Center for Biotechnology Information, which includes 514, 728, and 600 at the chromosome, contig, and scaffold levels, respectively (Figure 1A). Of these 1842 genome assemblies of insects, a total of 274 species are classified as invasive, of which 116, 32, and 126 have been classified at the chromosomal, contig, and scaffold levels, respectively (Figure 1A, Table S1). The release of these assemblies has rapidly increased in recent years: in 2021, the genome assemblies of 80 species were registered (Figure 1B). 

**Figure 2:**
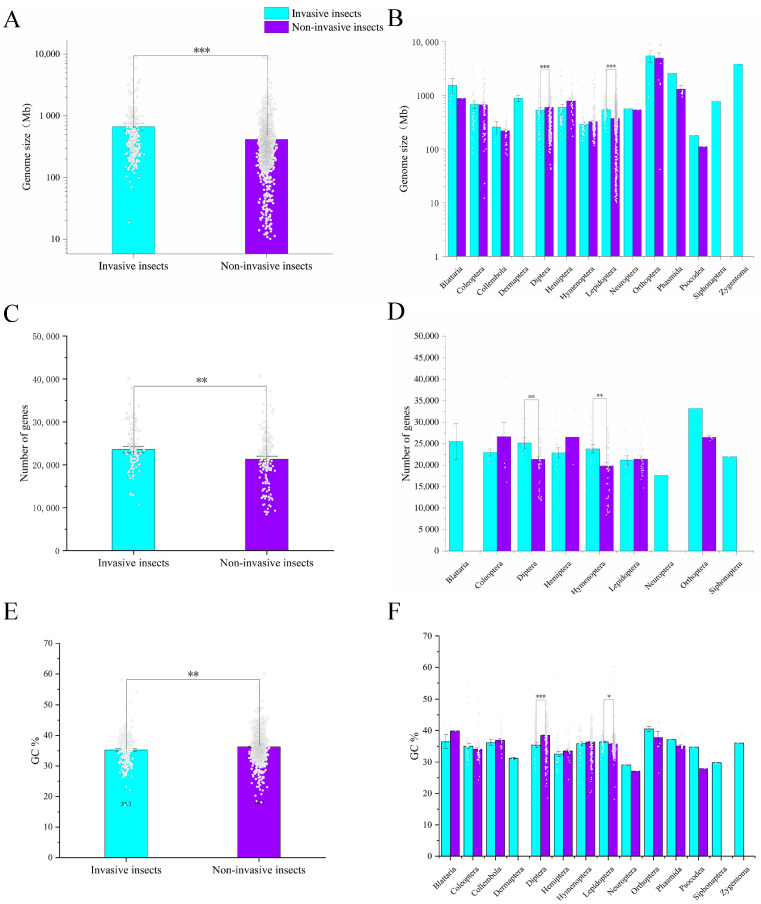
Comparison of genomic information between invasive and non-invasive insects. Comparison of average genome size (**A**) and genome sizes of different orders (**B**) between invasive and non-invasive insects. Comparison of the average number of genes (**C**) and the number of genes of different orders (**D**) between invasive and non-invasive insects. Comparison of average GC content (**E**) and GC content of different orders (**F**) between invasive and non-invasive insects. Asterisks indicate significant differences (*, *p* < 0.05; **, *p* < 0.01; ***, *p* < 0.001).

We compared the genome size of 274 invasive insects with that of other non-invasive insects and found that the genome size of invasive insects was significantly larger than that of non-invasive insects (Figure 2A, 670.94 vs. 417.60 Mb, *p* < 0.001), suggesting that invasive insects have more genomic resources to adapt to the environment. At different order levels, the genome size of invasive insects in Diptera and Lepidoptera were significantly higher than that of non-invasive insects, while there were no significant differences in genome size among other orders (Figure 2B). Among all of these invasive insects, the observed variation in genome size ranges from 18.61 Mb to 8.82 Gb (Table S1). The insect with the largest genome is *Schistocerca nitens* (8822.57 Mb), whereas that with the smallest is *Dactylopius coccus* (18.61 Mb). At the order level, orthopterans have the largest average genome (5.44 Gb), and psocodeans have the smallest average genome (178.25 Mb) (Figure 2B). In terms of the number of genes annotated in the genome sequence, the average number of genes of invasive insects was significantly higher than that of non-invasive insects (Figure 2C, 23,534 vs. 21,298, *p* < 0.01). At the order level, the number of annotated genes in invasive insects of Diptera and Hymenoptera was significantly higher than that in non-invasive insects (Figure 2D). Orthopterans also have the largest average gene number (Figure 2D). In terms of GC content, the average GC content of invasive insects was significantly lower than that of non-invasive insects (Figure 2E). Among them, the GC content of invasive insects of Diptera was significantly lower than that of non-invasive insects, while the GC content of Lepidoptera was significantly higher than that of non-invasive insects (Figure 2F).

The size of the genome and the number of annotated genes can reflect the adaptability of insects to the environment to some extent. From our statistical results, we found that invasive insects had significantly higher genome size and number of annotated genes than non-invasive insects (Figure 2), which often leads to the expansion of functional gene families associated with invasive traits (such as chemosensory and detoxification genes), and is likely to be the internal driving force for invasive insects to rapidly invade or outperform their native relatives. Some detailed mechanisms will be summarized in the following review.

## 3. Whole-Genome Sequencing Reveals Insect Invasion Mechanisms

Papers have been published on some of the invasive insect genomes (73/274, Table 1). From this literature, we summarized six types of invasion mechanisms of invasive insects that have been revealed based on genome sequencing, and we described the methods for the prevention and control of invasive insects.

### 3.1. Rapid Genetic Variation and Evolution of Invasive Populations

The evolution of organisms is an important internal driver of their adaptation to the environment. Generally, the more or the faster the genetic variation occurs, the easier the organisms adapt to the new environment. Transposable element (TE) proliferations are a pivotal class of mutations that facilitate genome expansion [92]. The Colorado potato beetle (*Leptinotarsa decemlineata*) achieves rapid evolutionary change through high-proportioned TEs (17% of the genome) [16]. More than half of the genome (51.7%) of the avian vampire fly (*Philornis downsi*) consists of TEs, which may be a key mechanism in its adaptive evolution [45]. The Asian Tiger mosquito (*A. albopictus*) genome contains all major TE groups, and repetitive sequences comprise approximately 68% of the genome, which is the most among all sequenced mosquito species [33,34]. Although *Cardiocondyla obscurior* has the smallest of the sequenced ant genomes, whose transposable element islands facilitate its adaptation to novel environments [67].

On the other hand, repetitive DNA sequences are emerging as a potential agent of the rapid adaptation of invasive species [93]. The genome of the German cockroach (*Blattella germanica*) contains 55% repetitive DNA, which is responsible for gene family expansion in *B. germanica* as well as in the drywood termite (*Cryptotermes secundus*) [19]. The genetic mechanisms of the adaptive capacity of the American cockroach (*Periplaneta americana*) to various environments may be attributed to highly repetitive sequences [20]. Repetitive sequences comprise 45.22% of the red turpentine beetle (*Dendroctonus valens*) genome, which is higher than that of other Coleoptera species [28]. The haploid western corn rootworm (*Diabrotica virgifera*) has the largest estimated genome (2.58 Gb) among the beetle species, which contains a higher proportion of repetitive sequence than that of *Tribolium castaneum* [29]. A total of 21.40% repetitive sequences were identified in the genome of the tephritid fruit fly (*Bactrocera tryoni*) [40]. The Russian wheat aphid (*Diuraphis noxia*) genome is the most AT-rich (70.9%) among the insect genomes sequenced up to date, with a widespread, extensive reduction in gene content potentially reflecting the effect of phytotoxic feeding on the genome formation of *D. noxia*, which, in turn, has expanded its host range [55,56].

In addition to TEs and repetitive DNA sequences, nucleotide diversity could be another driver of rapid insect adaptation [94]. For instance, *P. americana* owned abundant microsatellite simple sequence repeats that were higher than those of the other four blattaria insects [20]. *L. decemlineata* showed high nucleotide diversity (every 22 base pairs of coding DNA has a variable site), which is eight times higher than that of other beetles [16]. A genome-wide analysis of 37 flies’ (dipteran) species revealed large hidden variations in the sex chromosomes, suggesting that similar selective forces have shaped the unique evolution of the sex chromosomes [44]. Genome-wide association analysis of 16 invaded-range and six native-range population samples of the fruit fly (*Drosophila suzukii*) shows that the invasive status of *D. suzukii* can be demonstrated by the difference in SNP markers between the invasive species and the native species [42]. The genome-wide variation between the Asia II 1 and MEAM1 of whiteflies (*Bemisia tabaci*) may have led to the differences between the two species, including insecticide resistance and host plant range [49]. The results of comparative genomic analysis between convergently evolved obligate plant–ant mutualists and non-mutualists suggest that the mutualists’ genome has higher molecular evolution rates [70]. Genomic variation among refugial and colonized populations of the oriental fruit moth (*Grapholita molesta*) demonstrated adaptive genetic changes during the Quaternary [76].

From the results of the present review, invasive insects in general have greater TEs, repetitive DNA sequences, and gene nucleotide diversity than native species. If the genome size is relatively small, invasive insects can improve their ability to adapt to the environment through higher amounts of TEs. Previously, we had little understanding of the function of repetitive DNA sequences in organisms. More and more research on the function of repeats will help us better understand the genetic mechanism behind the faster genetic variation and evolutionary ability of invasive insects. Through the genome-level genetic variation mining and genome-level variation analysis of insects with different genetic backgrounds (such as invasive species and native species), it is not only helpful to elucidate the development and formation mechanism of invasive traits, but also to identify endemic species using molecular markers (such as molecular identification for invasive species).

### 3.2. Invasion History and Dispersal Path

Invasive insects pose a serious threat to agriculture, economies, and human health because they have superior migratory and spreading abilities, among others, resulting in an increasingly wide range of impacts, and the potential threat is unpredictable. As the peak accumulation of alien species has not yet occurred worldwide, which means existing studies and actions have largely been insufficient to reduce the continuous increase in the spread of alien species [5], the primary task is to determine the origin and occurrence regularity of invasive insects to effectively curb their cross-regional spread. Genome sequencing and sequence alignment analysis of species in different geographic populations can identify millions of genetic markers that can be used to distinguish between different individuals or geographic populations by calculating genotype frequency and population structure. For instance, genome-wide allele frequency differences indicated that L. decemlineata expanded into the Midwestern U.S. and Europe from the Great Plains [16]. Genome-wide markers were genotyped in 712 Asian long-horned beetles (*Anoplophora glabripennis*), individuals from their native distribution, showing six distinct population clusters among native *A. glabripennis* populations; most of the individuals from South Korea were substantially different from the *A. glabripennis* populations in China [26]. The complete genomes of *D. suzukii* collected from the continental United States showed that the population structure between western and eastern populations is strong, but no evidence has been found of any population structure between different latitudes, suggesting no broad-scale adaptations in response to differences in winter climates [43]. Genome resequencing of geographic *D. valens* populations in original and invaded regions suggested a large divergence in the North American population and revealed possible invasion and spreading routes in China [28]. Population sequencing of the aphid-like grape phylloxera (*Daktulosphaira vitifoliae*) revealed that the global invasion started up the Mississippi River in North America, spread to Europe, and then from there to other places around the world [52]. The results of a worldwide population analysis of the soybean aphid (*Aphis glycines*) suggested that the source of the invasive population in North America may be China or Korea [47]. The results of population genetic analysis with genome-wide microsatellites of the cotton mealybug (*Phenacoccus solenopsis*) revealed strong differentiation between three populations collected from the areas in China experiencing invasions [61]. The results of genomics and citizen science revealed that the small cabbage whitefly (*Pieris rapae*) spread from Eastern Europe to all continents except South America and Antarctica [81], and American populations started from a few foreign individuals [82]. A multi-population genetic analysis of the ancestry and mixing patterns of honeybees (*Apis mellifera*) suggested that *A. mellifera* originated in the Middle East or northeastern Africa [65]. The results of population genomics analysis indicated that the Chinese accessions of the fall armyworm (*S. frugiperda*) were derived from Africa rather than directly from the USA. [86]. The results of population genomic analysis of the light brown apple moth (*Epiphyas postvittana*) with a RAD-tag approach suggested that California moths probably came from Australia [75]. Genomes from the worldwide populations of the diamondback moth (*Plutella xylostella*) suggested that South America is the geographical origin of this species, which has undergone three major expansions around the world, largely driven by European colonization and global trade [83].

The traditional methods of analyzing the invasion history and diffusion path of invasive insects are generally completed through meteorological data of previous years, insect monitoring data, interception records of quarantine departments or customs, etc. combined with field investigation and sampling [95,96,97,98]. The acquisition and analysis of genetic markers at the genome level can measure the gene exchange among different geographic populations, and the invasion history and diffusion path of invasive insects with genetic molecular markers can be described by the evolutionary tree and related analysis [16,26,43]. The present review has found that this method has unparalleled advantages for studying the origin and dispersal routes of invasive insects: there is less interference from other external factors. However, the reliability of using molecular markers alone to reconstruct invasion history is controversial, as it can be affected by factors such as sampling range, type, and number of molecular markers used [99]. In the future, if the method based on the molecular markers is combined with the traditional method, it will be helpful to reveal the diffusion and spreading of the rule of invasive insects more systematically and accurately.

### 3.3. Rapid Adaptation to Different Host Plant Ranges

Rapid adaptation to host plants in new sites is the basis for the invasion, colonization, and spread of invasive insects, which are often better adapted to the same host plants than native insects, and some can even replace native species. Adaptation to host plants is mainly achieved through the detection, detoxification, and utilization of host plants.

Insects recognize host plants primarily through their chemosensory systems, especially their olfactory, gustatory, and ion-sensory systems. For example, *B. germanica* can detect many different chemosensory cues, which may be attributed to the largest number, by far, of ionic receptors (IRs) of any species investigated [19]. The chemosensory gene families in the stenophagous emerald ash borer (*Agrilus planipennis*) are smaller than those of the polyphagous wood-borer *A. glabripennis*, suggesting a correlation between the chemosensory gene content and host specificity in beetles [25]. The expansion of gustatory receptors facilitates the adaptation of *L. decemlineata* to plant feeding [16]. Several small lineage-specific chemoreceptor gene families have expanded in the wheat stem sawfly (*Cephus cinctus*), which may be related to its ability to adapt to new grasses, including wheat [68]. The replication of the olfactory receptor gene (OR3) has enhanced the ability of the codling moth (*Cydia pomonella*) to use kairomones and pheromones in locating host plants and mates [17]. Part of the reason for the highly polyphagous nature of the brown marmorated stink bug (*H. halys*) may be that it possesses the largest number of chemoreceptors ever observed in insects, especially gustatory receptors [57]. A remarkably high number (231) of genes coding candidate gustatory receptors (GRs) in *S. frugiperda* were observed compared with those of omnivorous lepidopteran species (45–74 genes), which probably facilitate host recognition [15,87]. The notably large increase in the number of gustatory receptors in the fall webworm (*Hyphantria cunea*) has helped it to colonize novel hosts [77,78]. Having the largest mosquito genome sequenced to date (1967 Mb), many gene families in *A. albopictus* involved in olfaction are considerably expanded [33].

Detoxification and digestion of toxic secondary metabolites secreted by host plants is the main internal reason for insect damage to plants. The presence of a relatively larger repertoire of CYP genes in *A. glycines* may be an adaptation to achieve its heteroecious life cycle [48]. The cytochrome P450 genes are much expanded in the Argentine ant (*Linepithema humile*) genome, which contributes to its unique features [69]. P450 clans 3 and 4 are expressed at almost all developmental stages, which may be an important mechanism through which *S. frugiperda* detoxifies plant xenobiotics [15]. Between two morphologically indistinguishable strains (corn strain and rice strain) of *S. frugiperda*, the substantial differences in the number of detoxification and digestion genes may account for the adaptation to different host plant ranges [87]. The families of genes involved in the detoxification of plant defense compounds in *P. xylostella* have remarkably expanded, underpinning its ability to adapt to the diversity of toxins in the host plant [84]. Divergent clades and large expansions in genes associated with detoxification (CYP4, CYP6, and CCE enzymes) were identified in the western flower thrips (*Frankliniella occidentalis*) [91]. 

The utilization of host plant nutrients and other components is also an important factor in insect host selection. *A. glabripennis* can feed on woody plants because its genome encodes a unique and diverse repertoire of enzymes that degrade major polysaccharide networks in plant cell walls [27]. The aspartyl protease gene families are expanded in the greenhouse whitefly (*Trialeurodes vaporariorum*) compared with *B. tabaci*, indicating its specific host-feeding characteristics relative to *B. tabaci* [64]. New genes for nectar and pollen use were found in *A. mellifera*, consistent with the ecological and social organization of the species [66]. A wide range of enzymes capable of degrading complex polysaccharides was identified in the coffee berry borer (*Hypothenemus hampei*), enabling a much more efficient use of its limited resources in green coffee seeds [31]. The lineage-specific gene family expansion in the pectinases has been remarkable in a walking stick (*Medauroidea extradentata*), which has contributed to its adaptability to its host plants [90].

Plants and insects have formed a unique way of interaction in the long process of coevolution. It is important to study their interaction mechanisms for developing effective anti-insect strategies. Genome sequencing can be used to discover the molecular biological basis of invasive insects in rapidly adapting and expanding host plants. On this basis, key interacting genes can be identified through further functional verification, which is helpful to explore new insecticidal targets and seek effective strategies for insect control by utilizing insect-plant interaction mechanisms.

### 3.4. Strong Environmental Adaptation

Analysis of gene–environment association identified 3049 candidate loci located in 874 genes linked to growth and reproduction, revealing the genetic bases for the adaptation of small hive beetles (*Aethina tumida*) to novel environments [21]. A rapid expansion of gene families and positively selected genes involved in metabolic capacity and signal transduction were identified in *D. valens*, which may have led to its rapid adaptation to its environment [28]. The expansions of the DDE transposase superfamily and unique gene families in the metabolism and defense response pathways in the oriental fruit fly (*Bactrocera dorsalis*) genome explain its environmental adaptability; the relatively high expression levels of heat shock proteins indicate an intrinsic mechanism for high-temperature adaptation regardless of environmental conditions [38]. The genetic diversity of the African fig fly (*Zaprionus indianus*) is relatively high compared with other closely related species, and the thermal niche of *Z. indianus* is the widest among the 13 species, which may have contributed to its range expansion [13]. More than four hundred olfactory receptors were identified in the fire ant (*Solenopsis invicta*) genome, which is the basis for *S. invicta* to adapt to a variety of complex environments [71]. The first sequenced geometrid genome of the winter moth (*Operophtera brumata*) revealed a novel circadian clock mechanism in female brachyptery, supported by large RDX-like gene families in the winter moth, revealing rapid genetic adaptation to climate change in winter moths [80]. We identified five core genes associated with the circadian rhythm in the peach fruit moth (*Carposina sasakii*) that are involved in regulating diapause-related adaptive transitions [74]. Genomic data from 75 sites spanning six continents proved that *P. xylostella* can largely tolerate future climates in most parts of the world and will continue to be a global pest beyond 2050 [85]. HSP genes, especially the Hsp70s group, have expanded in melon thrips (*Thrips palmi*) compared with other hemipterans, leading to changes in stress response phenotypes [100].

In addition to the need to adapt to host plants, insects also need to adapt to the outside climate, environment, and many other factors. The study of the interaction mechanism between insects and plants is thorough, but the study of the interaction mechanism between insects and the environment is relatively backward. By analyzing the correlation between genome sequencing and external environmental factors, it is helpful to systematically reveal the molecular mechanism of rapid adaptation of invasive insects to the environments. Some of the papers reviewed above have researched this aspect, and further functional research and verification of the identified genes and loci are still needed.

### 3.5. Development of Insecticide Resistance

In pest management, chemical control is still the most widely used insect pest control measure. Especially when invasive insects occur and other control methods are not immediately effective, chemical control can rapidly reduce the population density of insects without regional and seasonal restrictions, with effective results. However, similar to other insects, invasive insects tend to develop resistance to insecticides more quickly, posing a serious threat to sustainable control. Therefore, the resistance mechanisms of invasive insects to insecticides must be studied to achieve long-term pest control. The family of detoxification enzyme genes encoding receptors for bitter or toxic substances and detoxification enzymes has substantially expanded in *P. americana*, providing the ability to detect and detoxify multiple toxins [20]. Several gene families involved in insecticide detoxification and adaptation to different dietary resources showed an increased number of copies in the small hive beetle (*A. tumida*) [22]. A reference-grade genome assembly of mosquitos (*Anopheles stephensi*) highlighted the importance of genomic elements previously hidden in the biology of malaria mosquitoes, such as the discovery of 29 previously hidden members of the insecticide resistance gene and new candidate genetic elements for the widespread resistance observed in *A. stephensi*. This will accelerate the development of genetic control strategies for malaria transmission [36]. The expansion of detoxification genes families, such as P450s and UDP glucuronosyltransferases, may have contributed to the development of insecticide resistance traits and host range expansion in *B. tabaci* and may have facilitated species invasion in cropping systems under intensive management [23,50]. A recent copy number variant expansion of aconitase, ABC transporter, and esterase gene families, which may be related to insecticide resistance, was identified in the English grain aphid (*Sitobion avenae*) [62,63]. Orthologous associated with biological processes related to insecticide and dehydration resistances are expanded in mealybugs (*Maconellicoccus hirsutus*) [59]. A remarkable diversity of insecticide-resistant mutations was identified in global populations of *Myzus persicae* [60]. The results of comparative genomics analysis showed that the extremely expanded cytochrome P450 gene family and its copy number variation have increased insecticide resistance in *S. frugiperda* [15,88]. Several detoxification genes, such as AOX, UGT, and GST, in S. frugiperda responded to twenty-three insecticides [86]. Genome-wide association studies comparing insecticide-resistant and insecticide-susceptible strains of *C. pomonella* identified hundreds of single-nucleotide polymorphisms (SNPs) that may be associated with insecticide resistance, including three SNPs found in the CYP6B2 promoter [17]. In the resistant population of the navel orangeworm (*Amyelois transitella*), an exceptionally large genome region (including a cytochrome P450 gene cluster associated with pyrethroid and DDT resistance and the gene encoding the voltage-gated sodium para channel, with virtually no polymorphisms) was detected [73].

At present, the control of invasive insects is still largely dependent on insecticides, which not only cause environmental pollution, food safety, and other problems but also promote the development of high resistance in insects. How to develop new insecticides for the biological characteristics and specific targets of insect pests is a major trend in pesticide development. Many new insecticidal target sites can be identified based on genome sequencing. If combined with the study of important biological learning of insects, new insect control strategies can be developed through the study of important traits such as olfactory, gustatory, and ion sensory systems, mating, and reproduction of insects in addition to the direct target of insecticide resistance.

### 3.6. Synergistic Harm Caused by Invasive Insects and Endosymbiotic Bacteria

Gut microbes provide important functions for organisms, being involved in nutrition, metabolism, development, immunity, and reproduction [101,102]. Gut microbes also play a vital role in the invasion process, working with invading insects. For example, the inventory of metabolic genes in the genome of the pea aphid (*Acyrthosiphon pisum*) suggests extensive metabolite exchange, including the sharing of amino acid biosynthesis between the aphid and its symbiont, *Buchnera aphidicola* [46]. The Wolbachia endosymbiont from the common bed bug (*Cimex lectularius*) showed the highest number of genes were found to be differentially expressed after feeding on human blood, demonstrating a simultaneous and coordinated host/commensal response to hematophagous activity [51]. Two bacterial endosymbionts, the γ-proteobacterium *Moranella endobia* and the β-proteobacterium *Tremblaya prince*, were replaced several times over in the evolutionary history in the mealybug *Planococcus citri*, allowing the insect to survive on its nutrient-poor diet [103]. Some gut microbes and their metabolic pathways can assist in the metabolism and detoxification of their nutrients, which may be involved in the adaptation of *H. cunea* to its hosts [78].

In the coevolution process with symbiotic bacteria, the immune ability of invasive insects is also a crucial factor. The putative biosynthesis pathway of harmonine, which is one of the defense chemicals and a key factor in the strong immunity of the harlequin ladybird (*Harmonia axyridis*), was successfully unmasked using chromosome-level genome assembly [30]. The Asian citrus psyllid (*Diaphorina citri*) lacks genes for the IMD pathway and has a reduced immune capability, which may facilitate infections of citrus greening bacteria (*Liberbacter asiaticus*), probably through their relationship with microbial endosymbionts [53,54]. The detection of the expansion of antimicrobial peptide genes revealed that *H. hampei* has a wide range of antimicrobial defense mechanisms [31].

Given the important function of gut microbes in organisms, it is an exciting entry point to study the mechanism of gut microbes of invasive insects and propose to control insects by regulating intestinal flora. With more sophisticated and advanced sequencing and library-building strategies, the genomes of insect gut microbes can be isolated from the insect genome, which is essential for the study of gut microbes.

## 4. Using Genome Sequencing for Developing New Strategies to Control Invasive Insects

The ultimate goal of studying the invasion mechanisms of insects is to effectively control invasive insects. Having genomic resources will help with defining the molecular basis of the feeding habits and habitat characteristics of insect pests and identifying potential targets for pest management strategies. The results of genome sequencing can help with the development and establishment of invasive insect control methods in many aspects. For instance, the notable expansion of the gene families associated with chemoreception in *P. americana* shows the potential for new or refined applications based on semiochemicals to control this insect pest [20]. *L. decemlineata* has many gene duplications in the siRNA pathway, which may make it more sensitive to RNAi and aid in future efforts to develop RNAi as a pest management technology [16]. A high-quality assembled genome of the coconut rhinoceros beetle (*Oryctes rhinoceros*) provided 30 candidate dsRNA targets whose orthologs were experimentally validated as highly efficient targets for several species of beetles based on RNAi [32]. Understanding unique detoxification pathways and pathway members can help with determining which treatments might control an invasive parasite, *A. tumida*, of bee colonies, even in the presence of honey bees, which are notoriously sensitive to pesticides [22]. The high-quality *Aedes aegypti* reference genome doubled the number of known chemosensory ionizing receptors, a critical first step in the development of novel repellents, and revealed copy-number variations in the glutathione S-transferase gene involved in insecticide detoxification, which will motivate the search for new insecticides to prevent the development of resistance [32]. Analysis of the 50-year-old *A. albopictus* C6/36 genome provided insight into the utility of cell lines for viral transmission [35]. Genomic data related to sex determination, sex-specific gene expression, reproduction, and programmed cell death should considerably advance the efficiency of the sterile insect technique (SIT) for controlling the size and invasive properties of Mediterranean fruit fly (*Ceratitis capitata*) populations [41]. Genomic elucidations of improved Y-chromosome sequences in the olive fruit fly (*Bactrocera oleae*) will advance our understanding of the organization, function, and evolution of the Y chromosome and will hopefully provide access to insect sterility techniques [39]. With the combination of genome sequencing technology and classical genetics, the SNP genotypes of an individual melon fly (*Bactrocera cucurbitae*) could be inferred, thereby deducing pupae color phenotypes and understanding the genetic basis of inherited sex traits, which can be used to improve SIT in this species [37]. The availability of the *H. halys* genome sequence will facilitate the development of environmentally friendly biomolecular insecticides for use in conjunction with more traditional synthetic chemical-based control measures [57]. Based on the reference genome of the glassy-winged sharpshooter (*Homalodisca vitripennis*), a high-quality Wolbachia SP genome of nearly 1.7 MB was obtained, providing a useful resource for Wolbachia-mediated *H. vitripennis* control in its invasion range [58]. *S. frugiperda* in China is currently susceptible to genetically modified corn, which provides an important reference for the commercial planting of Bt corn in China [89]. The complete genome sequence of the European gypsy moth (*Lymantria dispar dispar*) may help identify the genetic determinants of flight control in this invasive insect and find potential molecular diagnostic markers to assist in identifying and intercepting *L. dispar* dispar in cargo containers [79]. High-quality assemblies of three invasive social wasps in the Vespula genus provide species-specific targets for novel control approaches, such as RNA interference, gene drivers, and the deployment of destructive viruses [72].

Based on genome sequencing, the characteristics of invasive insects can be systematically understood; on this basis, by combining the habits of each invasive insect, host interaction, traditional control methods, and so on. It is helpful to quickly and effectively develop targeted basic research and control strategies for different invasive insects. With further research, it is believed that more and more new control strategies against invasive insects will be developed.

## 5. Conclusions

At present, a variety of mechanisms used by insects to achieve invasion have been elucidated based on the genome sequencing of some invasive insects, including rapid genetic variation and evolution, revealing invasion history and dispersal paths, rapid adaptation to different host plant ranges, strong environmental adaptation, rapid development of insecticide resistance, and collaborative destruction involving endosymbiotic bacteria. Studies on invasion mechanism based on genome sequencing are crucial for the prevention and control of invasive insects. In the meantime, the specific genome sequencing results required for final prevention and control technologies need to be more comprehensive. Therefore, because many institutions are planning to sequence the genomes of more invasive insects, the sequencing needs to be closely integrated with prevention and control technologies, such as identifying key insecticidal targets for the development of novel repellents or RNAi-based control technologies, sterile insect technique (SIT), Wolbachia-mediated control, and so on. In addition to genome sequencing, other methods such as transcriptome and microbiome sequencing can be combined to further elucidate the invasion mechanisms.

## Figures and Tables

**Table 1:** Registered invasive insect genomes with the corresponding literature references.

Order	Scientific Name	Level	Total Length (Mb)	Gene Number	Release Date	References
Blattaria	* Blattella germanica *	Scaffold	2037.3	29,581	2018	[19]
	* Cryptotermes secundus *	Scaffold	1018.93	29,285	2018	[19]
	* Periplaneta americana *	Scaffold	3374.84	NA	2018	[20]
	* Zootermopsis nevadensis *	Scaffold	485.025	30,187	2014	
Coleoptera	* Aethina tumida *	Chromosome	259.984	21,401	2022	[21,22,23,24]
	* Agrilus planipennis *	Scaffold	353.073	22,159	2014	[25]
	* Anoplophora glabripennis *	Scaffold	706.969	20,632	2013	[26,27]
	* Dendroctonus valens *	Scaffold	322.407	NA	2022	[28]
	* Diabrotica virgifera *	Scaffold	2418.07	28,061	2018	[29]
	* Harmonia axyridis *	Chromosome	425.525	23,861	2021	[30]
	* Hypothenemus hampei *	Scaffold	162.571	NA	2020	[31]
	* Leptinotarsa decemlineata *	Scaffold	641.993	19,038	2013	[16]
	* Oryctes rhinoceros *	Scaffold	377.356	NA	2021	[24]
	* Rhynchophorus ferrugineus *	Scaffold	782.098	NA	2020	
Diptera	* Aedes aegypti *	Chromosome	1278.73	28,317	2017	[32]
	* Aedes albopictus *	Scaffold	2538.39	40,086	2019	[33,34,35]
	* Anopheles stephensi *	Chromosome	243.46	29,660	2020	[36]
	* Bactrocera cucurbitae *	Chromosome	244.9	13,286	NA	[37]
	* Bactrocera dorsalis *	Chromosome	530.327	30,102	2022	[38]
	* Bactrocera oleae *	Scaffold	484.947	33,727	2015	[39]
	* Bactrocera tryoni *	Chromosome	570.659	23,950	2021	[40]
	* Ceratitis capitata *	Scaffold	436.491	22,949	2013	[41]
	* Culex quinquefasciatus *	Chromosome	573.23	24,531	2020	
	* Drosophila suzukii *	Chromosome	268.012	25,540	2020	[42,43]
	* Liriomyza trifolii *	Scaffold	69.6986	NA	2015	[44]
	* Mayetiola destructor *	Scaffold	185.828	NA	2010	
	* Philornis downsi *	Scaffold	954.765	NA	2021	[45]
	* Zaprionus indianus *	Scaffold	197.261	NA	2021	[13]
Hemiptera	* Acyrthosiphon pisum *	Chromosome	533.649	27,913	2019	[46]
	* Aphis glycines *	Scaffold	308.064	18,358	2019	[47,48]
	* Bemisia tabaci *	Scaffold	615.017	22,737	2016	[23,49,50]
	* Cimex lectularius *	Scaffold	510.849	24,194	2014	[51]
	* Daktulosphaira vitifoliae *	Scaffold	282.6	NA	2022	[52]
	* Diaphorina citri *	Scaffold	485.705	24,730	2013	[53,54]
	* Diuraphis noxia *	Scaffold	395.074	17,476	2015	[55,56]
	* Euschistus heros *	Contig	1325.16	NA	2018	
	* Halyomorpha halys *	Scaffold	998.241	25,026	2014	[57]
	* Homalodisca vitripennis *	Chromosome	2305	31,163	2021	[58]
	* Lycorma delicatula *	Scaffold	2252	10,652	NA	
	* Maconellicoccus hirsutus *	Contig	189.239	NA	2018	[59]
	* Myzus persicae *	Scaffold	347.313	23,910	2016	[60]
	* Paracoccus marginatus *	Scaffold	191.208	NA	2016	
	* Phenacoccus solenopsis *	Chromosome	292.538	NA	2019	[61]
	* Piezodorus guildinii *	Chromosome	1205.42	NA	2022	
	* Sitobion avenae *	Scaffold	393.019	NA	2021	[62,63]
	* Trialeurodes vaporariorum *	Scaffold	787.484	NA	2020	[64]
Hymenoptera	* Apis mellifera *	Chromosome	225.251	23,471	2018	[65,66]
	* Cardiocondyla obscurior *	Scaffold	193.051	NA	2021	[67]
	* Cephus cinctus *	Scaffold	160.751	31,793	2014	[68]
	* Linepithema humile *	Scaffold	219.501	21,674	2011	[69]
	* Polistes dominula *	Scaffold	208.026	20,855	2015	
	* Pseudomyrmex gracilis *	Scaffold	282.776	23,473	2017	[70]
	* Solenopsis invicta *	Chromosome	378.102	30,910	2021	[71]
	* Vespula germanica *	Chromosome	205.789	NA	2021	[72]
	* Vespula pensylvanica *	Chromosome	179.37	23,842	2020	[72]
	* Vespula vulgaris *	Chromosome	188.205	NA	2021	[72]
Lepidoptera	* Amyelois transitella *	Scaffold	406.468	18,472	2015	[73]
	* Carposina sasakii *	Chromosome	399.04	NA	2020	[74]
	* Cydia pomonella *	Scaffold	772.892	NA	2018	[17]
	* Diatraea saccharalis *	Scaffold	453.235	NA	2020	
	* Epiphyas postvittana *	Scaffold	598.1	31,389	NA	[75]
	* Grapholita molesta *	Chromosome	517.71	NA	2022	[76]
	* Helicoverpa armigera *	Chromosome	356.686	28,010	2022	
	* Helicoverpa zea *	Chromosome	375.181	23,696	2022	
	* Hyphantria cunea *	Scaffold	510.512	NA	2018	[77,78]
	* Lymantria dispar dispar *	Scaffold	864.963	NA	2019	[79]
	* Operophtera brumata *	Chromosome	619.179	NA	2022	[80]
	* Pieris rapae *	Chromosome	256.37	20,497	2021	[81,82]
	* Plutella xylostella *	Chromosome	323.303	22,256	2022	[83,84,85]
	* Spodoptera frugiperda *	Chromosome	485.97	30,806	2020	[15,86,87,88,89]
Phasmida	* Medauroidea extradentata *	Scaffold	2593.36	NA	2018	[90]
Thysanoptera	* Frankliniella occidentalis * *Thrips palmi*	Scaffold Scaffold	274.99 237.845	23,356 27,217	2014 2020	[91] [37]

## Data Availability

Not applicable.

## Supplementary Materials

The following supporting information can be downloaded at: https://www.mdpi.com/article/10.3390/ijms24098004/s1.
